# Polysaccharides from *Ganoderma Sinense* - rice bran fermentation products and their anti-tumor activities on non-small-cell lung cancer

**DOI:** 10.1186/s12906-021-03346-7

**Published:** 2021-06-10

**Authors:** Wei Han, Hongjuan Chen, Lin Zhou, Haijie Zou, Xiaohong Luo, Bo Sun, Xuhui Zhuang

**Affiliations:** 1 Academy of National Food and Strategic Reserves Administration, Beijing, 100037 P. R. China; 2 China National Tranditional Chinese Medicine Co., Ltd, Beijing, 100077 P. R. China

**Keywords:** Rice bran polysaccharides, *Ganoderma sinense* fermentation, Anti-tumor activity, H1299 non-small-cell lung cancer, In vivo and in vitro

## Abstract

**Background:**

Non-small-cell lung cancer (NSCLC) accounts more than 80% of the lung cancer cases. Polysaccharides in rice bran and its fermentation products have been proven to suppress many cancers. However, the report on inhibiting NSCLC is few. In this paper, the polysaccharides with suppression activity to H1299 NSCLC in the fermentation products of full-fat rice bran and defatted rice bran were studied in vitro and in vivo.

**Method:**

Polysaccharides (GSRBPs) were extracted from *Ganoderma sinense* – full-fat rice bran (GS-FRB) and *Ganoderma sinense* – defatted rice bran (GS-DRB) fermentation products. The structure information of the GSRBPs was studied using HPLC analysis. The anti-tumor activities on H1299 NSCLC of GSRBPs in vitro study was performed using MTT method. The in vivo studies use BALB/c-nu nude mice as H1299 NSCLC bearing mice.

**Result:**

All the polysaccharides contained two fractions, GSFPS-1 and GSFPS-2. The molecular weight and the ratio of GSFPS-1 and GSFPS-2 were different in GS-FRB and GS-DRB. At the earlier state of fermentation, all polysaccharides were composed of D-glu, D-man, D-xyl and L-ara with certain molar ratios. But at the latter stage, polysaccharides in GS-FRB were composed of D-glu, D-man, D-xyl, L-ara and D-fru, while these in GS-DRB only composed of D-glu and D-man. In the in vitro study, the IC50 of RBS and GSRBPs was as GS-DRB-11 (40.62 μg/mL), GS-FRB-9 (43.82 μg/mL), GS-DRB-7 (48.08 μg/mL), RBS (49.56 μg/mL), GS-DRB-9 (49.91 μg/mL), GS-DRB-13 (51.89 μg/mL), GS-FRB-11 (53.75 μg/mL), GS-FRB-7 (56.84 μg/mL), GS-DRB-13 (60.63 μg/mL) from small to large. In the in vivo study, the H1299 NSCLC inhibition rate (InRa) of RBS and GSRBPs were GS-DRB-11 (86.81%) > GS-DRB-9 (86.01%) > GS-FRB-9 (84.88%) > GS-DRB-7 (82.21%) > GS-DRB-13 (78.04%) > RBS (76.06%) > GS-FRB-13 (65.44%) > GS-FRB-11 (64.70%) > GS-FRB-7 (27.87%). The GSFPS-2 area percent was negatively correlated to the IC50 and was positively correlated to the InRa. This means the GSFPS-2 had much higher anti-tumor activity than GSFPS-1.

**Conclusion:**

GSFPS-2 had higher anti-tumor activities, and the lipid in the rice bran has a decisive effect on the structures of polysaccharides produced by fermentation. Therefore, GSRBPs could be considered as potential novel agents to suppress H1299 non-small-cell lung cancer.

**Supplementary Information:**

The online version contains supplementary material available at 10.1186/s12906-021-03346-7.

## Background

Lung cancer, characterized by high incidence, mortality and poor prognosis, is one of the most malignant tumors [1]. Among the lung cancer cases, more than 80% are identified as non-small-cell lung cancer (NSCLC) and it is responsible for over one million mortalities worldwide each year [2, 3]. Although the chemotherapy and radiotherapy are used in curing cancer patients, they often show serious side effects [4]. Many natural products such as polysaccharides and some secondary metabolites have been proven to be effective in inhibiting the growth of NSCLC lines [5–7]. H1299 is derived from lymph node human NSCLC stable cell line and can divide indefinitely. In addition, H1299 cells have a homozygous deletion of the TP53 gene which was the first tumor suppressor gene has been identified [8]. Therefore, H1299 cells lack the tumor suppressor P53 protein which is expressed by the TP53 gene. It makes H1299 (p53-null) cell more sensitive to some compounds [9].

Rice (*Oryza sativa* L.) bran, one of the main by-products in the rice milling process, is rich in fat, protein, carbohydrates and many other active compounds [10]. Among these valuable compositions, rice bran polysaccharides (RBS) have drawn amount of attention from natural product researchers as they have been found to inhibit the growth of various types of tumors such as gastrointestinal cancers, [11] mammary tumors, [12] and Lewis lung carcinomas [13]. MGN-3/Biobran, the bio-modified RBS fermented using shiitake mushrooms has been proven to have activities to suppress many cancers such as leukemic cells, myeloma and colorectal cancer in vitro, in vivo and in clinic studies [14]. However, there were few studies about the bioactivities of RBS and its fermented products on H1299 NSCLC. *Ganoderma sinense* (GS) is an important and representative edible and medicinal fungus, which contains anti-tumor polysaccharides [15]. Therefore, the present work attempted to study the polysaccharides from the fermentation products of *Ganoderma sinense* - full-fat rice bran (GS-FRB) and *Ganoderma sinense* - defatted rice bran (GS-DRB) at different fermentation stages. Different polysaccharides were compared in the aspects of structure and anti-tumor activity. In this study, BALB/c-nu mice were selected as H1299 NSCLC tumor-bearing mice for they showed hairless skin and defective development of the thy mic epithelium, which makes the mice phenotypically lack hair and absent of T cells [16].

## Methods

### Materials and reagents

*Ganoderma sinense* (CGMCC: 5.69) was acquired from the Institute of microbiology, Chinese Academy of Sciences. The full-fat rice bran (FRB) and defatted rice bran (DRB) were purchased from Henan Siwei Biotech Co., ltd (Zhengzhou, China). a-Amylase (50 U/mg), gluco-amylase (70 U/mg) and trypsin (4000 U/g) were purchased from Solarbio (Beijing, China). Monosaccharide standards were purchased from Sigma-Aldrich (Shanghai, China). H1299 cell line was provided by ATCC (USA). Dulbecco’s Modified Eagle’s Medium (DMEM), serum free medium (SFM) and Fetal Bovine Serum (FBS) were provided by GIBCO Company (USA), BALB/c-nu nude mice (female, 20 ± 2 g) were provided by Institute of Laboratory Animal Sciences, Chinese Academy of Medical Sciences (Beijing, China). Phosphate buffered saline (PBS), Dimethyl sulfoxide (DMSO) and 3-(4,5-dimethylthiazol-2-yl)-2, 5-diphenyltertrazolium bromide (MTT) were purchased from Solarbio (Beijing, China).

### Preparation of fermentation product

GS fungal mycelia were cultured in potato dextrose agar (PDA) medium and then were inoculated in 50 mL of a liquid potato dextrose (PD) medium containing 1% glucose, 0.3% potato extract, 0.2% KH_2_PO_4_ and 0.1% MgSO_4_·7H_2_O and cultured for 9 days at 26 °C, 200 r/min. The mycelia cultured on liquid PD media were inoculated into 50 g sterilized FRB and DRB medium respectively and were cultured at 28 °C. The fermentation products were harvested at several different time points (0, 7, 9, 11 and 13 days) and then dried at 60 °C for 24 h.

### Preparation of polysaccharides

Dried fermentation products (5 g) were extracted with petroleum ether and 70% hot ethanol for 3 times, respectively. The residues were extracted with hot water for 3 times at a ratio of 1:10 (w/v). The extracts were collected by filtration. Starch and proteins were removed from the extracts by α-amylase, glucoamylase and pepsin at 55 °C. 4 fold volumes of 95% ethanol were added into the supernatant and the mixtures were stored at 4 °C for 12 h. The precipitates were collected by centrifugation at 4000 r/min for 30 min and subsequently dissolved in distilled water. The crude *Ganoderma sinense* –rice bran polysaccharide (GSRBP) samples were obtained by freeze-dried of the supernatant. The yields of the polysaccharides were determined by phenol-sulfuric acid method.

### Determine of molecular weight distribution

The molecular weight (Mw) distributions of the RBS and GSRBP were determined by using a HPGPC (Waerst e2695, USA) equipped with a refractive index detector (RID). Waters Ultrahydrogel™ 1000 column (7.8 × 300 mm) and Waters Ultrahydrogel™ 250 column (7.8 × 300 mm) were used in series. Sample solutions (20 μL) was injected and run with 0.1 mol/L NaNO_3_ aqueous solution at 0.8 mL/min as mobile phase. The column-oven temperature was 40 °C. The standard curve was established by using Dextran for molecular weights (from Macklin, Mw: 4320 Da, 12,600 Da, 60,600 Da, 110,000 Da and 289,000 Da) as the standards. The molecular weight of each composition was calculated by contrast with the retention time of polysaccharides reference standard.

### Monosaccharide composition analysis

10 mg RBS and GSRBPs were hydrolyzed with 2 mL 1.0 M trifluoroacetic acid (TFA) at 105 °C in a sealed-tube for 2 h. The TFA was removed using decompression evaporation accompanied. The hydrolysates were solved in 10 mL distilled water to obtain 1.0 mg/mL sample hydrolysis solutions. Samples (10 μL) were injected into the HPLC (Waters e2695) equipped with an evaporative light Scattering Detector (ELSD). Waters XBridge Amide column (3.5 μm, 4.6 × 150 mm) was used. The Molar ratios of the monosaccharides were calculated using standard curves with D-glucose (D-glu), D-mannose (D-man), D-xylose (D-xyl), L-arabinose (L-ara) and D-fructose (D-fru) as standards. The HPLC conditions: Column temperature was 35 C; mobile phase was acetonitrile – water added with 0.2% triethylamine (80: 20) with the flow rate of 0.25 mL/min; gas pressure was 40 psi and drift tube temperature was 70 °C in ELSD.

### Assay of in vitro antitumor activity

H1299 cells were inoculated into 96-well plates with 1000 cells/well, cultured at 37 °C and 5% CO2 for 24 h. The RBS and GSRBP solutions with the concentration of 0.01 μg/mL, 0.1 μg/mL, 1 μg/mL, 10 μg/mL and 100 μg/mL were prepared by PBS solution with pH 7.0, respectively, and then they were added into the 96-well plate containing H1299 NSCLC cells. DMEM culture medium (100 μL) was added to the blank wells as the 0 μg/mL group. There were 6 wells in each group. The cells were cultured in an incubator for 24 h. Then, 100 μL DMEM and 15 μL MTT (10 mg/mL) were added in to the wells and then left to stand for 4 h in a cell culture tank. DMEM containing MTT was discarded, washed with PBS until the cleaning solution was colorless, 100 μL DMSO was added, mixed with a little shock for 30 min, and OD490 was detected. The antitumor activities in vitro of RBS and GSRBPs were determined by the cell viability of H1299 NSCLC cells.

### Assay of in vivo antitumor activity

All the protocols of in vivo activity experiment were approved by the Academy of National Food and Strategic Reserves Administration Committee on the Use of Experimental Animals (Protocol number: LA2018–1012), and conform to the “Laboratory animal control ordinance (2017)” and “Guidelines for ethical review of experimental animal welfare (GB/T 35892-2018)” of China.

The in vivo experiment was operated in the SPF animal facility of Academy of National Food and Strategic Reserves Administration. Adult BALB/c-nu nude mice (female, 8 weeks old, 20 ± 2 g) were maintained in sterile conditions at 22–26 °C, 55 ± 2% humidity, with 12 h light-dark cycles and ad libitum access to SPF grade mice maintained diet (Shanghai changshuo biotechnology co. LTD). Mice were housed in Basf polysulfone boxes (420 × 250× 230 mm, 5 mice per box), All boxes contained sterile wood shavings as bedding. Animal welfare related assessments and interventions were conducted during the experiment, including morphology, physiology, behavior, dietary intake, water consumption and general health status. Before the start of the experiment, none of the mice received any experiment or any additional treatment.

H1299 cells suspension (0.1 mL, 1 × 10^6^ cells/mL for each mice) was subcutaneously inoculated into the right axilla of 50 BALB/c-nu mice. The growth of the tumors was observed every other day. At 6 d after H1299 tumor cells inoculation, the mice were randomly divided into 10 groups with 5 mice each group: one negative control (physiological saline) group, one positive control (40 mg/kg RBS treated) group, and 8 experimental (40 mg/kg GSRBPs treated) groups. Dose design of RBS and GSRBPs as well as their concentrations were by reference to the pretest. RBS and GSRBPs were dissolved in physiological saline, and then administered orally once daily. The body weight and tumor volume of nude mice were measured weekly post H1299 cells inoculation. Tumor volume was obtained following the formula of: tumor volume (mm^3^) = 0.52 × major axis × (minor axis)^2^. The major axis and the minor axis were collected using digital vernier calipers. After 42 days administration, all the nude mice were euthanized by cervical dislocation and solid tumors were excised for tumor weight determination. InRa calculated by the following formula: InRa (%) = [(A − B)/A] × 100, A is the average tumor weight of the negative control group; B is the average tumor weight of treated groups. The tumor tissue samples were embedded with paraffin, and then detected by the routine hematoxylin-eosin (HE) staining for pathological test.

### Data analysis

The data were expressed as the mean ± standard deviation (SD) of triplicates obtained from 2 to 3 independent assays. Data in all the bioactive assays were statistically evaluated by ANOVA and Tukey’s test using DPS statistic package and Microsoft Excel. The differences observed in statistical comparison were considered significant when *P < 0.05 and **P < 0.01.

## Results

### Structural differences of the polysaccharides between RBS and GSRBPs during fermentation

The yields of the RBS were 2.33% ± 0.07 and 4.32% ± 0.05%, respectively in FRB and DRB. In the GS-FRB fermentation products, the yield of GSRBPs increased first (4.67% ± 0.12 and 7.64% ± 0.16% in 7d and 9d, respectively) and then decreased (7.00% ± 0.26 and 5.15% ±0.15% in 11d and 13d, respectively). In the GS-DRB fermentation product, the order of GSRBP yield was as follows: GS-DRB-11 (9.67% ± 0.18%), GS-DRB-13 (9.44% ± 0.12%), GS-DRB-7 (8.33% ± 0.30%) and GS-DRB-9 (6.33% ± 0.12%).

**Fig. 1:**
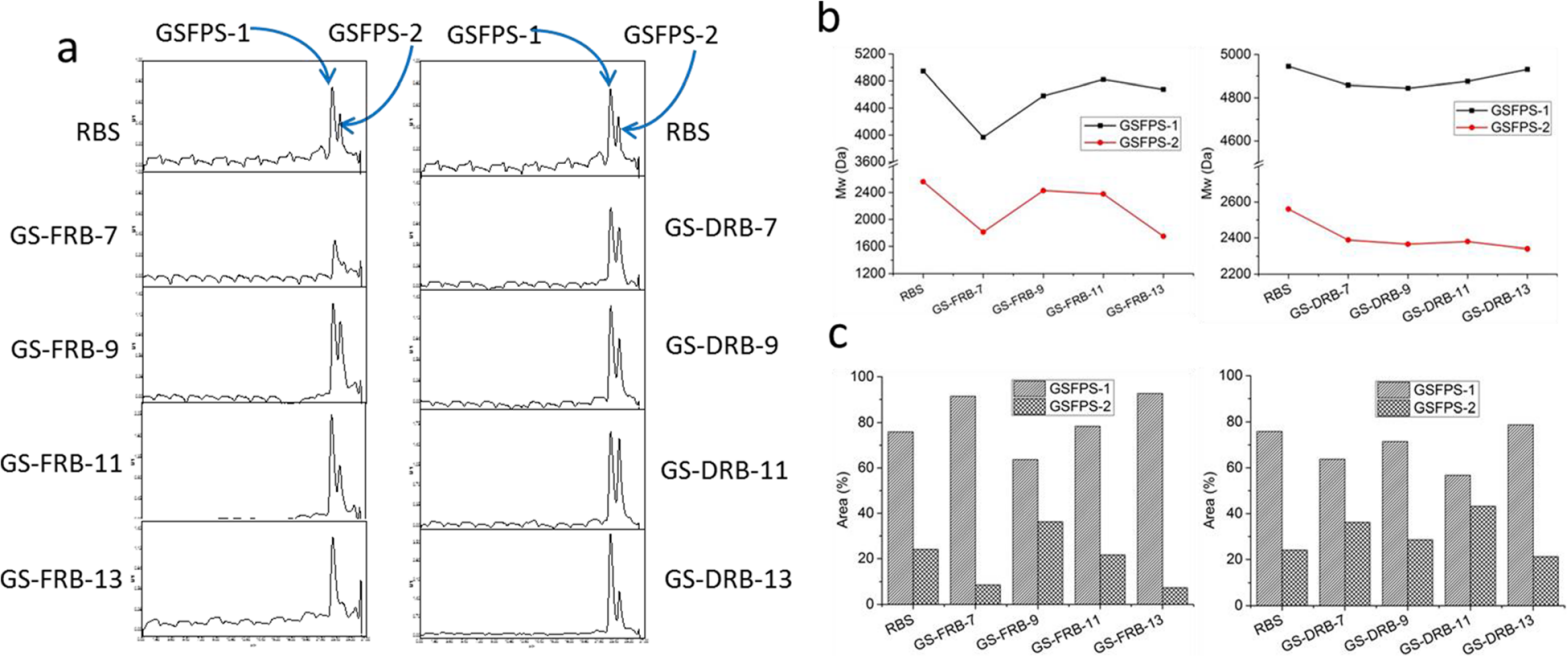
The HPGPC chromatograms of RBS and GSRBPs (**a**), their molecular weights (**b**) as well as the area percentage of GSFPS-1 and GSFPS-2 (**c**). Both RBS and GSRBPs are composed of two fractions, GSFPS-1 and GSFPS-1 respectively. The ratio of the two fractions and their Mws were also changed during fermentation

Both RBS and GSRBPs were composed of two polysaccharide fractions, GSFPS-1 with a larger molecular weight (Mw) and GSFPS-2 with a smaller Mw (Fig. 1a). The Mws of the GSFPS-1 and GSFPS-2 of RBS were 4944.84 Da and 2560.64 Da, respectively (Fig. 1b). Both the Mws of GSFPS-1 and GSFPS-2 were decreased in GSRBPs at the 7th fermentation day in GS-FRB (3966.19 Da and 1811.77 Da) and GS-DRB (4858.50 Da and 2388.11 Da) as Fig. 1b shows. Both the Mws of GSFPS-1 and GSFPS-2 changed with the fermentation time prolonged in GS-FRB and GS-DRB. In GS-FRB, the Mw of GSFPS-1 increased into 4582.52 Da and 4820.99 Da, respectively at 9d and 11d and then declined to 4677.11 Da at 13d. Similar to GSFPS-1, the Mw of GSFPS-2 in GS-FRB also increased at 9d (2428.83 Da), but then declined to 2378.03 Da and 1749.05 Da at 11d and 13d, respectively. The Mws of GSFPS-1 in GS-DRB hardly changed at 7d, 9d and 11d (4858.50 Da, 4844.82 Da and 4875.64 Da), and it only increased on the 13d (4930.92 Da). The Mws of GSFPS-2 in GS-DRB hardly changed in the fermentation duration and they were 2388.11 Da, 2364.67 Da, 2379.71 Da and 2339.81 Da, respectively in 7d, 9d 11d and 13d. The area percentage of GSFPS-1 or GSFPS-2 is the ratio of its peak area in HPGPC to the sum of the areas of GSFPS-1 and GSFPS-2, and the result summarized as Fig. 1c shows. The percentage of GSFPS-1 and GSFPS-2 was 75.9 and 24.1%, respectively in RBS. The area ratios of GSFPS-1 and GSFPS-2 were obvious differences between GS-FRB and GS-DRB. In GS-FRB, the ratio of GSFPS-1:GSFPS-2 was increased to 91.43%:8.57% at 7d. However, it decreased to 63.73%:36.27% at 9d and then increased to 78.36%:21.64 and 92.68%:7.32% at 11d and 13d, respectively. In GS-DRB, The ratio of GSFPS-1:GSFPS-2 was decreased to 63.79%:36.21% at 7d and then went up to 71.46%:28.54% at 9d. It was 56.75%:43.25 and 78.78%:21.22% at 11d and 13d, respectively. The Mws and ratios of the two fractions in GS-FRB and GS-DRB were quite different along the fermentation procedure.

The monosaccharide compositions of the RBS and GSRBPs were as Table 1 shows. According to HPLC analysis, RBS was composed of D-glu, D-man, D-xyl and L-ara with a molar ratio of 30.5:8.0:5.4:8.7. At the earlier stage of the fermentation (7d and 9d), all the GSRBPs were composed of D-glu, D-man, D-xyl and L-ara both in GS-FRB and GS-DRB. However, at the latter stage of fermentation (11d and 13d), the composition evolves in different directions. In GS-FRB, the GSRBPs were composed of D-glu, D-man, D-xyl, L-ara and D-fru, while these in GS-DRB only composed of D-glu and D-man. DRB was obtained by removing the lipid from FRB. The differences of the monosaccharide compositions indicate the lipid in rice bran might influent the structures of the polysaccharides produced in the fermentation.

**Table 1:** Monosaccharide compositions of the polysaccharides from RBS, GS-FRB and GS-DRB. At beginning, all the GSRBPs were composed of D-glu, D-man, D-xyl and L-ara both in GS-FRB and GS-DRB. But at the late stage of fermentation, the GSRBPs were composed of D-glu, D-man, D-xyl, L-ara and D-fru in GS-FRB, while these in GS-DRB only composed of D-glu and D-man

polysaccharides	Molar ratio
RBS	D-glu: D-man: D-xyl: L-ara = 30.5: 8.0: 5.4: 8.7
GS-FRB-7	D-glu: D-man: D-xyl: L-ara = 29.3: 7.0: 5.4: 7.9
GS-FRB-9	D-glu: D-man: D-xyl: L-ara = 61.6: 6.2: 7.0: 10.6
GS-FRB-11	D-glu: D-man: D-fru: D-xyl: L-ara = 33.0: 5.7: 4.1: 7.0: 9.1
GS-FRB-13	D-glu: D-man: D-fru: D-xyl: L-ara = 52.4: 5.9: 6.8: 6.5: 9.9
GS-DRB-7	D-glu: D-man: D-xyl: L-ara = 19.2: 6.7: 5.4: 6.8
GS-DRB-9	D-glu: D-man: D-xyl: L-ara = 23.5: 6.2: 7.5: 7.2
GS-DRB-11	D-glu: D-man = 12.7: 5.9
GS-DRB-13	D-glu: D-man = 10.6: 6.2

### Anti-tumor effect of the RBS and GSRBPs in vitro

**Fig. 2:**
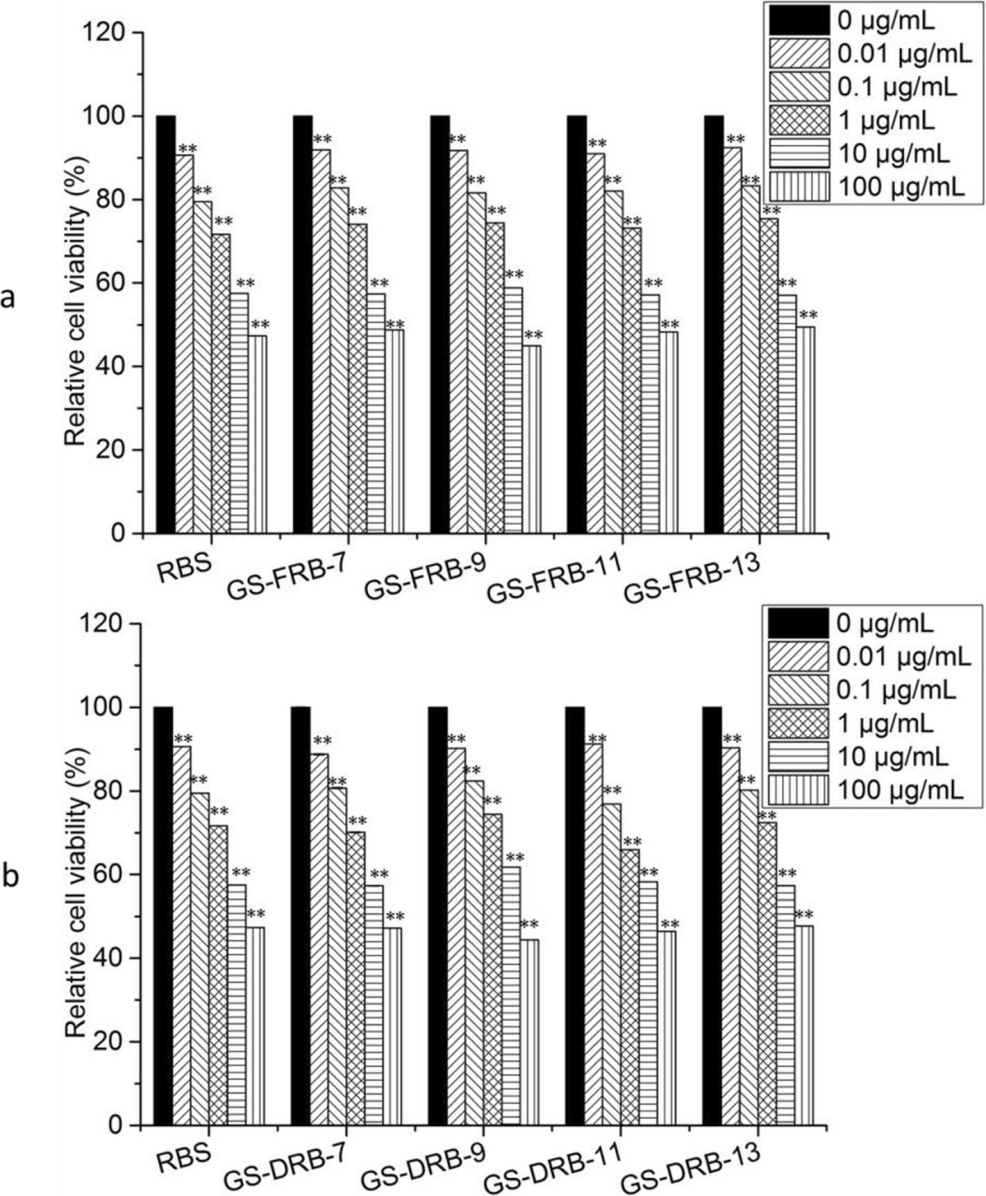
In vitro effect of polysaccharides extracted in GS-FRB (**a**) and GS-DRB (**b**) at different concentrations on H1299 NSCLC cell viability by MTT assay. All the polysaccharides could inhibit the viability of H1299 NSCLC. Each value represents the mean ± SE (*n* = 6). ∗ *P* < 0.05, ∗∗ *P* < 0.01 as compared to 0 μg/mL

MTT test showed that the cell survival rate of H1299 NSCLC decreased linearly with the increase of concentrations of RBS and GSRBPs in the range of 0.01 μg/mL ~ 100 μg/mL (Fig. 2). When the concentration was 100 μg/mL, the cell survival rates of RBS, GS-FRB-7, GS-FRB-9, GS-FRB-11 and GS-FRB-13 was 47.35, 48.76, 44.97, 48.25 and 49.47%, respectively. Their 50% inhibitory concentration (IC50) was 49.56 μg/mL, 56.84 μg/mL, 43.82 μg/mL, 53.75 μg/mL and 60.63 μg/mL, respectively. That means the GS-FRB-9 has the best anti-tumor activities and the GS-FRB-13 has the lowest. In GS-DRB groups, the cell survival rates of RBS, GS-DRB-7, GS-DRB-9, GS-DRB-11 and GS-DRB-13 was 47.35, 47.23, 44.36, 46.44, 47.71%, respectively at the concentration of 100 μg/mL. Their 50% inhibitory concentration (IC50) was 49.56 μg/mL, 48.08 μg/mL, 49.91 μg/mL, 40.62 μg/mL and 51.89 μg/mL, respectively.

The order of the IC50 of RBS and GSRBPs was as GS-DRB-11, GS-FRB-9, GS-DRB-7, RBS, GS-DRB-9, GS-DRB-13, GS-FRB-11, GS-FRB-7, GS-DRB-13 from small to large.

### Anti-tumor effect of the RBS and GSRBPs in vivo

**Fig. 3:**
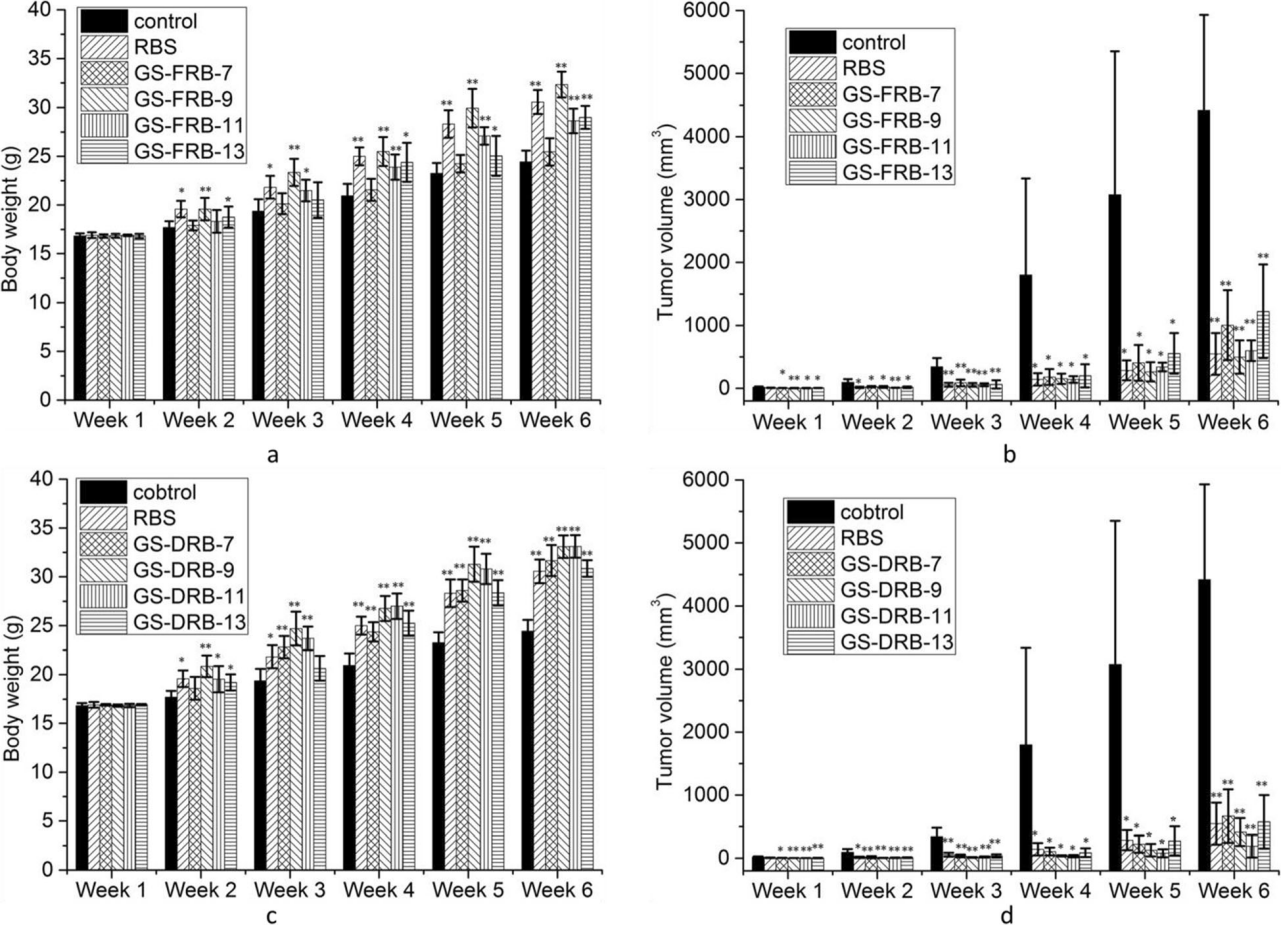
In vivo effect of polysaccharides extracted in GS-FRB (**a** and **b**) and GS-DRB (c and d) on mice body weight (a and c) and tumor volume (**b** and **d**). The difference in body weight gain between the sample group and the control group increased with the time of administration prolonged. Sample groups had a good inhibitory effect on the growth of H1299 NSCLC. Each value represents the mean ± SE (*n* = 5). ∗ *P* < 0.05, ∗∗ *P* < 0.01 as compared to control group at the corresponding time

Figure 3 shows the body weights and tumor volumes of H1299 NSCLC bearing mice after oral administration of control, RBS and GSRBPs from GS-FRB and GS-DRB. No adverse side effects or unintentional deaths were observed during the treatment period and all mice were in good physical and mental condition (6 weeks). In addition, RBS and GSRBPs treated mice showed significant growths of bodyweight gain (Fig. 3a and c). Most polysaccharides showed significant effect in retardation of solid tumor development as early as within 2 weeks at the treatment period (Fig. 3b and d). After 6 weeks, the tumor volume in the control group was 4416.62 mm^3^ ± 1513.73 mm^3^. The tumor volume in RBS group was 547.95 mm^3^ ± 332.07 mm^3^ (12.41 ± 7.52% of the control group). The order of the tumor volumes in GSRBP groups were as follows: GS-DRB-11 (4.26% ± 4.05% of the control group), GS-DRB-9 (9.35% ± 4.98% of the control group), GS-FRB-9 (11.26% ± 5.99% of the control group), GS-DRB-13 (13.03% ± 9.56% of the control group), GS-FRB-11 (13.53% ± 3.69% of the control group), GS-DRB-7 (15.12% ± 9.59% of the control group), GS-FRB-7 (22.72% ± 12.58% of the control group), and GS-FRB-13 (27.67% ± 16.80% of the control group). All of them showed significant differences (*P* < 0.01). The data of the tumor volumes of control, RBS and GSRBPs groups can be found in Supplementary data. GS-DRB-11, GS-DRB-9 and GS-FRB-9 could inhibit the tumor growth of H1299 NSCLC better than RBS. On the whole, the anti-tumor activities of GSRBPs of GS-DRB were better than these in GS-FRB. Among the GSRBPs, GS-FRB-13 which showed the lowest anti-tumor activity had the largest GSFPS-1:GSFPS-2 value (92.68%:7.32%), while that of GS-DRB-11 with the highest bioactive was the opposite (56.75%:43.25%).

**Fig. 4:**
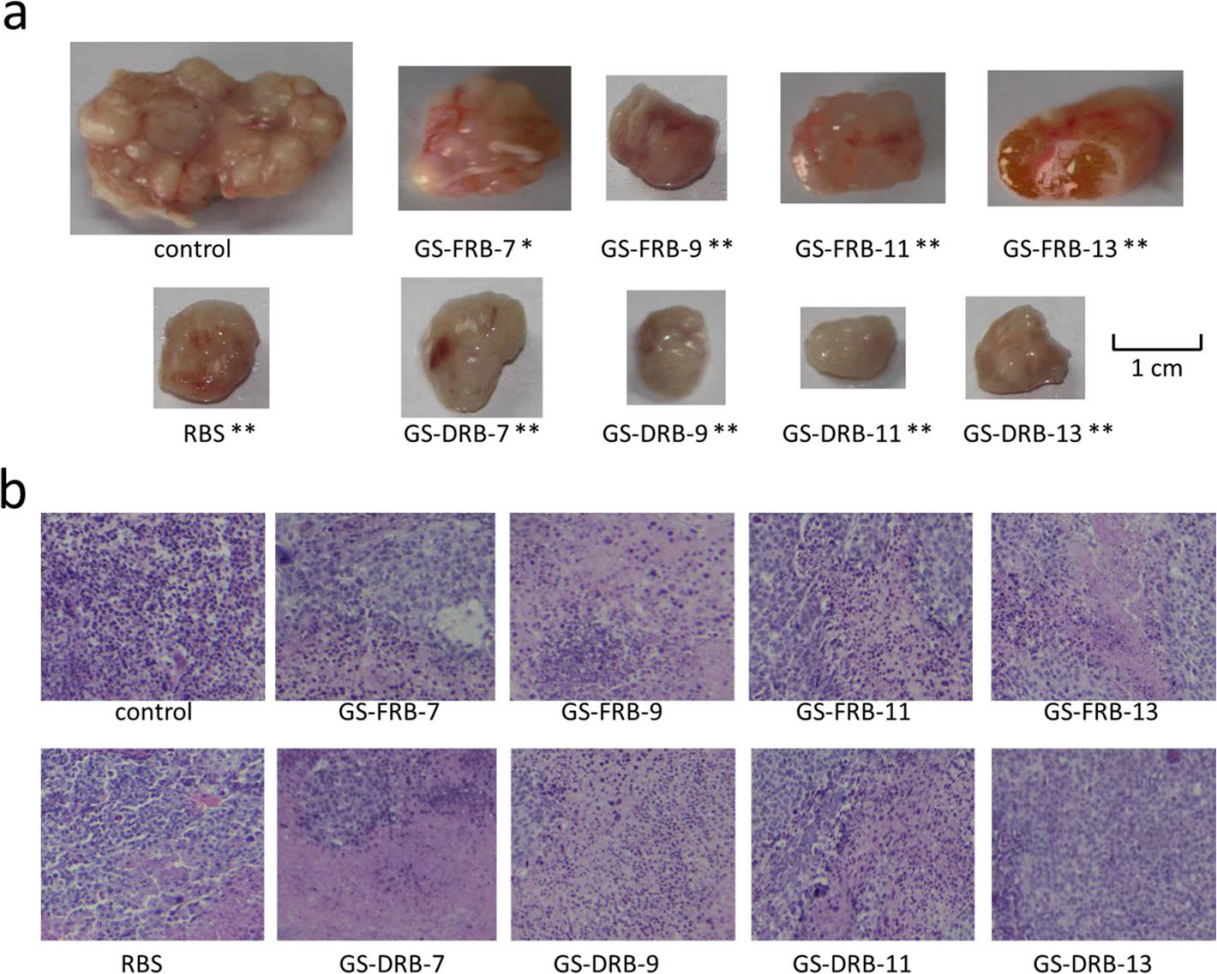
Photographs of solid tumors (**a**) and their representative images of HE-stained sections. (**b**) from H1299 NSCLC bearing mice after the 6-week treatment trial. The tumors in the sample groups were much smaller than those in the control group. In the pathological sections of the control group, the tumor nuclei (dark purple) were large, while the tumor cells were apoptosis significantly in the sample group. ∗:*P* < 0.05, ∗∗:*P* < 0.01 of the tumor weight as compared to control group

Photographs of the tumor isolated from mice in sample groups after experiment demonstrated significant tumor regressions as compared to control group (Fig. 4a). The tumor weight in control group was 2.70 ± 0.70 g. The order of the tumor weight in sample groups were as follows: GS-DRB-11 (0.36 ± 0.09 g, the InRa 86.81%) < GS-DRB-9 (0.38 ± 0.10 g, the InRa 86.01%) < GS-FRB-9 (0.41 ± 0.12 g, the InRa 84.88%) < GS-DRB-7 (0.48 ± 0.11 g, the InRa 82.21%) < GS-DRB-13 (0.59 ± 0.10 g, the InRa 78.04%) < RBS (0.65 ± 0.20 g, the InRa 76.06%) < GS-FRB-13 (0.93 ± 0.07 g, the InRa 65.44%) < GS-FRB-11 (0.95 ± 0.15 g, the InRa 64.70%) < GS-FRB-7 (1.94 ± 0.40 g, the InRa 27.87%). Compare to the control group, the tumor weight of the GS-FRB-7 group was statistically significant (*P* < 0.05) and that of other groups were very significant (*P* < 0.01). The order of the tumor weight in sample groups after experiment was not exactly the same to the tumor volume after 6 weeks’ treatment. The reason is that the tumor volume was calculated by the measured major axis and the minor axis of solid tumor on living mice, and it might bring mistakes.

Histopathological sections were examined to present the apoptotic cells of solid tumors in mice post-treated with RBS and GSRBPs (Fig. 4b). The cells in control group had large nucleoplasm, clear nuclear margin, and showed irregular shapes, which accorded with the characteristics of solid tumor. The tumor cells in the sample groups showed apoptotic. It was speculated that the polysaccharides might be toxic and cytotoxic to tumor cells.

**Fig. 5:**
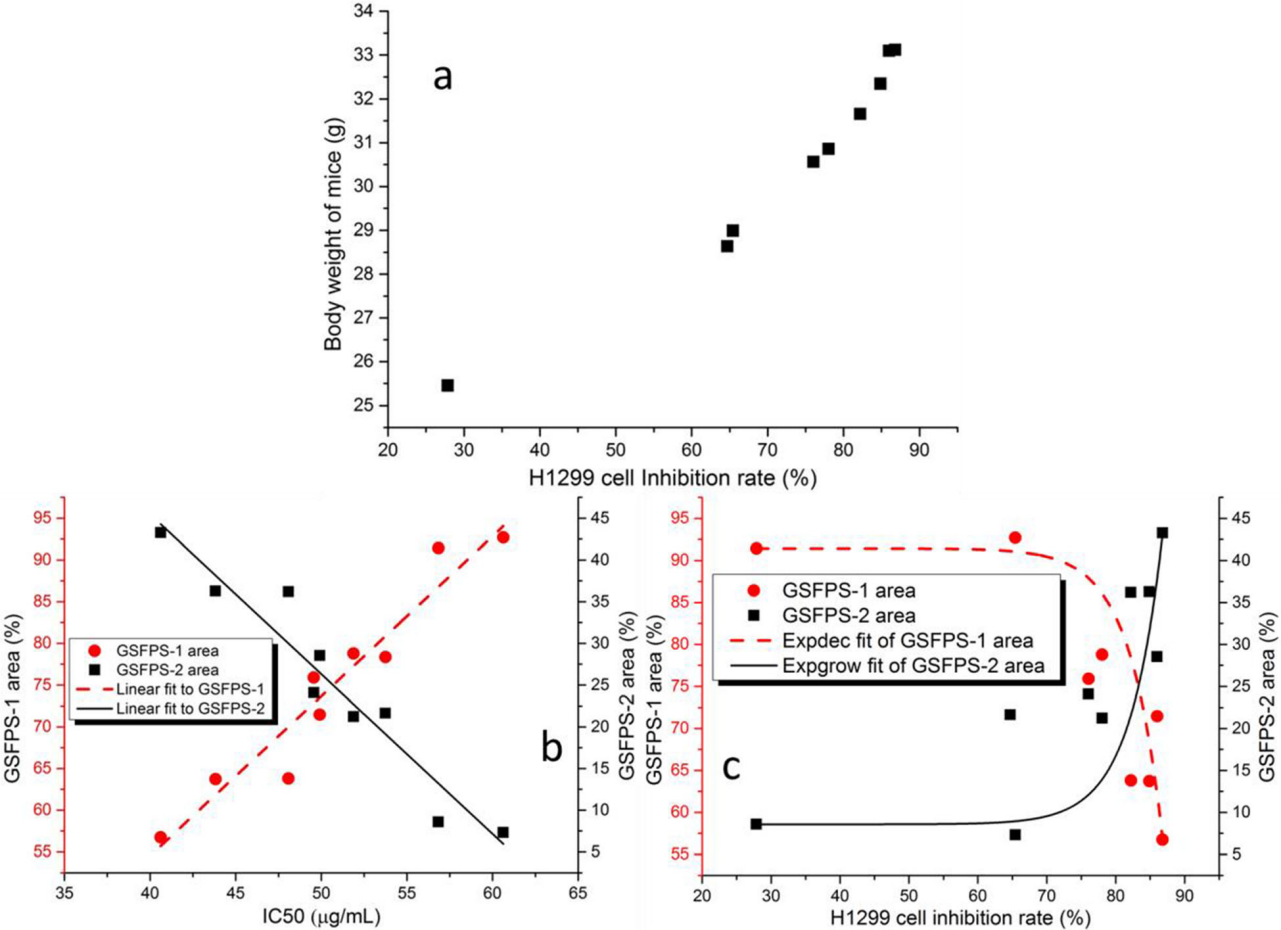
(**a**) H1299-NSCLC InRas at different body weights in nude mice. The InRa of H1299 NSCLC was positively correlated with the body weight of the tumor-bear mice. (**b**) IC50 at different GSFPS-1 areas (red circle) and GSFPS-2 areas (black squares). It was positive correlate between InRa of H1299 NSCLC and the GSFPS-1: GSFPS-2 ratio. (**c**) H1299-NSCLC InRa at different GSFPS-1 areas (black squares) and GSFPS-2 areas (black pentagram). The correlation between InRa of H1299 NSCLC and the GSFPS-1: GSFPS-2 ratio was negative

The InRa of H1299 NSCLC seems to be positively correlated with the body weight of the tumor-bear mice (correlation coefficient was 0.9666), as Fig. 5a shows. To maintain the energy and biosynthetic precursor demands of proliferation, tumor cells have to influence the organism metabolism, such as accelerated rate of aerobic glycolysis [17, 18], alterations in levels of Krebs cycle intermediates [18, 19], and activation of the pentose phosphate pathway [18], which increased nutrient and lipid consumption and then in turn affects the body weight. The polysaccharide with higher anti-tumor activities could suppress the proliferation of the tumor, thus, there were higher body weight gain in these groups.

Figure 5b shows the correlations of the GSFPS-1 area and GSFPS-2 area to the corresponding IC50. The areas of GSFPS-1 in RBS and GSRBPs were linearly correlated with the corresponding IC50 with the correlation equation of Area_GSFPS-1_ = 1.92 × IC50–22.11, R^2^ = 0.9160. The areas of GSFPS-2 in RBS and GSRBPs were also linearly correlated with the corresponding IC50 with the correlation equation of Area_GSFPS-2_ = − 1.92 × IC50 + 122.11, R^2^ = 0.9160. The IC50 was positively correlated with the GSFPS-1 area and negatively correlated with the GSFPS-2 area.

Figure 5c shows the correlations of the GSFPS-1 area and GSFPS-2 area to their corresponding InRas of H1299 NSCLC. The areas of GSFPS-1 in RBS and GSRBPs were exponentially correlated with the corresponding InRa with the correlation equation of Area_GSFPS-1_ = − 3.30 × 10^− 7^ × exp. (InRa/4.70), R^2^ = 0.2441. The areas of GSFPS-2 in RBS and GSRBPs were also exponentially correlated with the corresponding InRa with the correlation equation of Area_GSFPS-2_ = 8.57 + 1.24 × 10^− 4^ × exp.[(InRa-27.87)/4.70], R^2^ = 0.0929. The InRa was negatively correlated with the GSFPS-1 area and positively correlated with the GSFPS-2 area. Both Fig. 5b and c indicated that the GSFPS-2 had much higher anti-tumor activity of H1299 NSCLC than that of GSFPS-1. It’s difficult to judge whether GSFPS-1 has anti-tumor activities based on the data above. Therefore, the separation and anti-tumor activity of the two polysaccharide fractions still need to be further studied.

## Discussion

The chemical structure of polysaccharides is diversity. Its biological activities depend on its structural characteristics. Therefore, numerous studies are devoted to modifying RBS by various means in order to find new high active polysaccharides. Many work found that the bio-activities of the modified RBS can be improved significantly using physical, chemical or biological means [12, 20–22]. Bio-modification of RBS is applied widely based on its economical. Most of the previous studies focused on the bio-modification of the RBS using the enzymes generated by bacteria. MGN-3/Biobran is a bio-modified RBS using the carbohydrate hydrolysing enzymes from shiitake mushrooms [23]. mRBPSs [21], polysaccharides with the weight-average molecular weight of 10^4^–10^5^ Da, was obtained from the modification of RBS using the intracellular enzymes from *Grifola frondosa* and could strengthen the natural killer cells’ cytotoxicity to K562 cells. However, the polysaccharides [20] obtained from the fermentation product of *Grifola frondosa* - rice bran water extract has the weight-average molecular weight of 10^2^–10^3^ Da. Thus, the polysaccharides from the fermentation product of microbe - rice bran might be different from the modified RBS using the glycosidase from this microbe. However, few consider the polysaccharides from the fermentation product of edible fungi - rice bran. The advantages of this bases on its convenience and high yield; moreover, due to the complexity of the organisms, it may obtain new polysaccharide that cannot be discovered by simple enzymatic reactions.

In the whole fermentation process, the Mw, monosaccharide composition, the area ratio of GSFPS-1 and GSFPS-2 as well as their anti-tumor activities in the fermentation products of GS-FRB and GS-DRB were quite different, which means the lipid in the rice bran had significant influence in the structures of the polysaccharides in the fermentation procedure. We haven’t found any report about the effect of lipid on the structures of polysaccharides in fermentation process. However, the organism is a complex system, and the difference of culture conditions has a great influence on the enzyme and its metabolites. Therefore, the polysaccharide in GS-RB fermentation product cannot be simply equivalent to the hydrolysis reaction of microbial glycosidase to the RBS.

## Conclusion

Polysaccharides extracted from byproducts of grains have been proven to possess anti-tumor activities. The method of producing high-bioactive polysaccharides using fermentation with byproducts of grain has many advantages. In this study, the polysaccharides from the products of GS-FRB and GS-DRB were discussed in detail in terms of structure and anti-H1299 NSCLC activity. All the polysaccharides had two fractions of GSFPS-1 and GSFPS-2. The polysaccharides from GS-FRB and GS-DRB possessed different structures and GSFPS-1: GSFPS-2 ratio. The anti-tumor activities of polysaccharides from GS-DRB were much higher than that from GS-FRB at the corresponding time point, and depended on the GSFPS-1: GSFPS-2 ratio. It suggested that GSFPS-2 had higher anti-tumor activities, and the lipid in the rice bran has a decisive effect on the structures of polysaccharides produced by fermentation.

## Supplementary Information

**Additional file 1.** Supplementary data accompanies this paper named.

## Data Availability

All data generated or analysed during this study are included in this published article and its supplementary information file [Supplementary data.xlsx].

## Abbreviations

NSCLC: Non-small-cell lung cancer
RB: Rice bran
RBS: Rice bran polysaccharides
FRB: Full-fat rice bran
DRB: Defatted rice bran
GS-FRB: *Ganoderma sinense* – full-fat rice bran
GS-DRB: *Ganoderma sinense* – defatted rice bran
GSRBP: Ganoderma sinense –rice bran polysaccharide
DMEM: Dulbecco’s Modified Eagle’s Medium
SFM: Serum free medium
FBS: Fetal Bovine Serum
PDA: Potato dextrose agar
PD: Potato dextrose
RID: Refractive index detector
TFA: Trifluoroacetic acid
ELSD: Evaporative light Scattering Detector
HE: Hematoxylin-eosin
SD: Standard deviation
Mw: Molecular weight
D-glu: D-glucose
D-man: D-mannose
D-xyl: D-xylose
L-ara: L-arabinose
D-fru: D-fructose
PBS: phos-phate buffered saline
MTT: 3-(4,5-dimethylthiazol-2-yl)-2, 5-diphenyltertrazolium bromide.
DMSO: Dimethyl sulfoxide
IC50: 50% inhibitory concentration
InRa: Inhibition rate
